# Eminence of Leader Humility for Follower Creativity During COVID-19: The Role of Self-Efficacy and Proactive Personality

**DOI:** 10.3389/fpsyg.2021.790517

**Published:** 2022-01-07

**Authors:** Farwa Asghar, Shahid Mahmood, Kanwal Iqbal Khan, Madeeha Gohar Qureshi, Mahendra Fakhri

**Affiliations:** ^1^Department of Commerce, The Islamia University of Bahawalpur, Bahawalpur, Pakistan; ^2^Institute of Business Management and Administrative Sciences, The Islamia University of Bahawalpur, Bahawalpur, Pakistan; ^3^Institute of Business & Management, University of Engineering and Technology, Lahore, Lahore, Pakistan; ^4^Pakistan Institute of Development Economics, Islamabad, Pakistan; ^5^Department of Business Administration, Telkom University, Bandung, Indonesia

**Keywords:** leader humility, follower creativity, social sustainability, COVID-19, self-efficacy, proactive personality

## Abstract

The purpose of this study is to understand how leader humility effectively stimulates follower creativity in the workplace during the coronavirus disease 2019 (COVID-19) scenario. Relying on social cognitive and social information processing theories, this study investigates how leader humility cultivates follower self-efficacy and follower creativity. Furthermore, it explores an intervening mechanism of follower self-efficacy and examines a moderating role of leader proactive personality. The hypothesized model is empirically tested by collecting the data from 405 employees and 87 managers working in the banking sector of Pakistan. The results indicate that leader humility is positively related to follower self-efficacy and follower creativity, which improve the organization’s innovation climate and an environment for social sustainability. Follower self-efficacy is also significantly related to follower creativity. The mediation analysis shows that follower self-efficacy mediates the relationship between leader humility and follower creativity. Additionally, leader proactive personality moderates the relation between follower self-efficacy and follower creativity. This study highlights the importance of leader humility for creativity and extends the literature by explaining the role of self-efficacy. Furthermore, the findings may assist the policymakers in how a humble leader heightens employee creativity and social sustainability in COVID-19.

## Introduction

In a dynamic environment, organizations have to deal with several challenges for their survival and maintaining a competitive advantage over their rivals. However, coronavirus disease 2019 (COVID-19) has been proven to be an infuriating global challenge (Khan et al., 2021). Initially, the COVID-19 pandemic seriously influenced the economy of different countries rapidly. Although many countries took different steps to control this uncertain situation, still COVID-19 spread drastically and affected human life. Management and policymakers have paid considerable attention to the COVID situation. This virus develops anxiety and a depressed environment that adversely affect the employees’ health. Leaders can control the anxiety and depression of employees as they directly interact with them. In this situation, a leader can play a positive role. Leader behavior can motivate employees for new and valuable ideas, which lead to creativity, innovation, and social sustainability.

There is a need to find and implement innovative solutions to mental health (Kapoor and Kaufman, 2020). Although organizational literature has witnessed the emerging interest of scholars in exploring the relation between leader behavior and employee creativity (Gu et al., 2020; Song et al., 2020). Although researchers have focused on different leadership styles that may influence creativity, but little attention has been paid to leader humility. Researchers argued that how humble leaders augmented follower creativity. Empirical evidence demonstrated that leader humility positively influences firm and team performance (Chiu et al., 2016). Even though humble leaders exert positive and motivational behavior, this study mainly focuses on how humble leaders’ positive attributes influence creativity in COVID-19 anxiety.

It becomes more critical for organizations to focus on leadership styles to control this dynamic situation like COVID-19. COVID-19 influences personal life as well as global organizations. It also increases the need for effective leadership during COVID-19 (Sergent and Stajkovic, 2020). Leader humility is a leadership style that positively influences employees in pandemics (Septiandari et al., 2021). It is also found that leader humility positively influences followers’ job satisfaction and work engagement (Owens et al., 2013). Leadership styles influence the creativity in an organization, such as transformational leadership, empowering leadership, and humility leadership (Wang et al., 2017). The social information processing theory suggests that leader humility increases employees’ creativity (Wang et al., 2017). Recently, it is articulated that more research is required to examine the effect of leader humility in enhancing follower creativity (Wang et al., 2017; Mao et al., 2019).

In a dynamic environment, *creativity* also plays a vital role in the survival of an organization. Innovation arises from creative ideas (Amabile et al., 1996). Academia and practitioners are interested in identifying the factors that enhance creativity (Bogilović et al., 2017). Leadership is one of the crucial factors concerning creativity. For instance, recent studies show a significant influence of leadership styles on employee creativity and team creativity (Gu et al., 2020; He et al., 2020). Follower creativity produces unique, novel, and helpful procedures, products, and services (Amabile et al., 1996). Researchers have devoted substantial attention to creativity and its antecedents and the factors influencing follower creativity. Researchers have extensively focused on the individual factors that affect creativity, such as psychological states, traits, thinking styles, self-concepts and identity, values, knowledge, and abilities for creativity (Anderson et al., 2014). Moreover, followers who perceive the workplace novel events and critical events are more likely to be improvised and creative (Chen et al., 2020). Although events play a vital role in stimulating creativity, but motivational factors are also important, especially in COVID-19 crises. Motivation-oriented factors provoke followers to employ in creative endeavors by incorporating their attributes, abilities, and skills (Cai et al., 2020).

Based on the social cognitive theory, human beings have the capability for observational learning, which enables them to develop their skills and knowledge under the influence of modeling (Bandura, 2001). Modeling gives cues about the rules for innovative and generative behavior (Bandura, 2001). An individual who observes their creative coworker’s behavior learns creative skills and strategies (Zhou, 2003). Employees can observe and learn either from a leader or a coworker, or a team member. Further, scholars suggested that wellbeing can be improved by continuously engaging in creative activities during the pandemic (Kapoor and Kaufman, 2020). However, there is a lack of intervening mechanisms that may strengthen the impact of leader humility on followers’ behavior (Mao et al., 2019).

Extensive studies has been focused on self-efficacy as an intervening mechanism (Chen et al., 2001; Mao et al., 2019). Self-efficacy is the trust of one person in the other persons’ abilities and skills to successfully organize and implement the work-related tasks to achieve organizational goals (Bandura, 1997). The social cognitive theory explains it as the confidence of an individual in their coworkers to perform a task successfully (Bandura, 2001). Individuals believe that they are competent and see more opportunities in risk decisions and tasks (Krueger and Dickson, 1994). Self-efficacy beliefs play an integral role in motivation, such as self-regulation (Bandura, 2001). It can be noted that COVID-19 causes mental distress. However, self-efficacy is still proven to be an intervening mechanism under the COVID-19 situation (AlZgool et al., 2020).

However, scholars claimed that leader humility augments follower creativity and follower self-efficacy. However, it is also essential how actively leaders respond to a dynamic environment. Proactive people engage in behavior beyond their direct requirements (Yang et al., 2020). They can assist their organization other than the assigned duties and exert a high-level effort for the organization in a vibrant situation. They also scan the opportunities and change the organization’s work environment (Bateman and Crant, 1993). In this regard, researchers have paid considerable attention to proactive behavior. For instance, they develop a climate that supports innovation (Xu et al., 2019). Creativity and innovation have been considered as vital components for the effectiveness and success of organizations (Anderson et al., 2014). Followers can emulate leader behavior as leaders are role models for them. Taking together, the proactive personalities of leader and follower enhance job satisfaction (Zhang et al., 2012). Proactive personality is positively related to motivational resources even in COVID-19 crises (Yi-Feng Chen et al., 2021). Prior studies have recommended that a leader’s proactive personality can be a boundary condition (Chiu et al., 2016; Wang and Liang, 2020).

**FIGURE 1:**
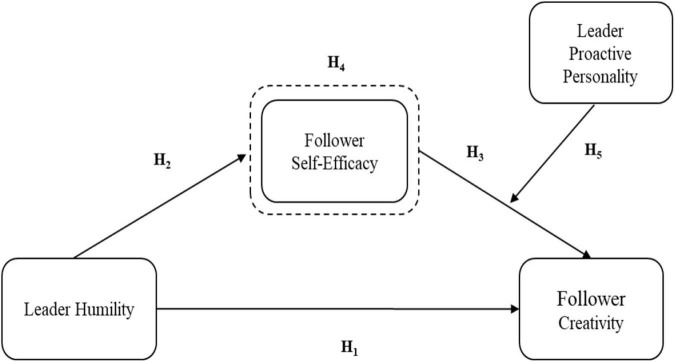
Theoretical framework. Dotted box represent the mediating path.

Based on social cognitive and social information processing theories, we investigate the impact of leader humility on creativity and response to the recent calls (Mao et al., 2019). This empirical study attempts to test a theoretical framework (Figure 1). We aimed to examine the interrelationship between leader humility, follower self-efficacy, and follower creativity. Moreover, the study examines an intervening mechanism of follower self-efficacy and the moderating role of a leader’s proactive personality.

## Theoretical Background and Hypotheses

According to the social cognitive theory, personal factors (i.e., cognitive and behavioral patterns) influence one another. The social cognitive theory is embedded in triadic reciprocal causation in cognitive processes (Bandura, 2001). Mutual causation in the workplace gives opportunities to learn for both the leader and follower. A humble leader also shows a willingness to learn and enhances the role of the follower and capability beyond one-self. It allows followers to exhibit their efficacy as a humble leader appreciates others’ strength. Cognitive processes are about brain activities and exercise a determinative influence (Bandura, 2001). Leaders try to influence their followers by integrating the strengths and weaknesses of the followers. Specifically, leader humility enhances follower self-efficacy (Mao et al., 2019).

The social information processing theory proposes that supervisors and coworkers are the leading providers of social cues (Griffin, 1983). Social cues from leaders have a positive impact on the follower behavior and perception. This theory also stated that the employee perception and responses about work-related tasks were affected by the changes in task objectives and information cues by leaders. Based on this theory, we posit that the link between leader humility and employee creativity depends on the social cues from the leader. Social cues motivate followers to be more creative and innovative. This theory also supports our proposed hypothesized model as social cues can cause followers to perceive the workplace more favorable (Griffin, 1983). Scholars argue that social information influences perceptions, attitudes, and behaviors in the workplace (Salancik and Pfeffer, 1978). Likewise, social cues can cause followers to perceive the workplace more favorable (Griffin, 1983). We argue that humble leader cues influence the outcomes of followers, such as leader humility enhances follower creativity (Wang et al., 2017). We expect that social cues allow the follower to show his capability to perform a different task during COVID-19. The follower’s confidence in performing a different task may lead to being more creative and unique.

### Leader Humility and Follower Creativity

Leader humility is an interpersonal characteristic that helps leaders cope with others in a social context by showing self-awareness (interaction with others), appreciating others’ contribution, strength, and teaching ability, i.e., unique ideas and feedback (Owens et al., 2013). A humble leader influences the group performance through constructive interpersonal processes in collective humility and collective regulatory focus (Owens and Hekman, 2016). Contextual, cultural, and personal factors affect creativity (Liu et al., 2016; Zhou et al., 2017). Creativity is a unique and productive idea. The novel idea depends upon the context that cues either good or bad being novel (Zhou et al., 2017). Indeed, individual creativity has been mainly focused. In recent years, team creativity has also received considerable attention (He et al., 2020). Specific leader behaviors significantly affect the subordinate perceptions of leader support that improve subordinate creativity (Amabile et al., 2004). Shared leadership and transformational leadership promote team creativity (Song et al., 2020). Supportive leader promotes creativity among followers due to the incorporation of intrinsic motivation and positive behavior (Gu et al., 2020). Moreover, task complexity is also associated with creativity (Sia and Appu, 2015).

Many empirical findings stated that leaders amplify follower creativity through intrinsic motivation. Extant research explored that a leader significantly affects the behavior of a follower behavior. As a particular leader’s behavior strongly influences the perception and reactions of subordinates, which affects subordinates’ creativity (Amabile et al., 2004). Different leadership styles can enhance creativity in the organization, such as transformational leadership (Shin and Zhou, 2007), humility leadership (Wang et al., 2017), and shared leadership (He et al., 2020; Song et al., 2020). Although we argue that different factors influence creativity, it leads to innovation, high performance, and suitability of organization. Nevertheless, some contingent factors limit this favorable effect of creativity. For instance, Gong et al. (2013) contend that a positive relation between creativity and firm performance is contingent on riskiness orientation, organization size, and realized absorptive capacity.

According to the social information processing theory, leader social cues can affect the follower motivation toward creativity (Griffin, 1983). We examine leader humility through the perspective of the social information processing theory. Leader humility is an interpersonal characteristic that helps leaders cope with others in a social context by showing self-awareness (an interaction with others), appreciating others’ contribution and strength, and teaching ability, i.e., unique ideas and feedback (Owens et al., 2013). A leader’ modeling of teachability allows the follower to be creative. Due to teachability in a humble leader, the follower can give a novel and unique idea. The environment is dynamic; for survival, there is a need for creativity. There is vast advancement in technology and increasing specialization. We argue that a humble leader can enhance follower creativity through teachability in the pandemic. Moreover, followers with humble leaders are more inclined to give valuable and new ideas. Shreds of evidence have shown that a humble leader positively affects follower creativity (Wang et al., 2017).

This study focuses on creativity by integrating social context as leader humility pays attention to teaching ability and self-enhancement of others. It also enriches the follower creativity (Wang et al., 2017). Drawing on social information processing theory, scholars argue that leader humility increases employees’ creativity (Wang et al., 2017). Leader humility positively influences employee accountability during COVID-19 (Septiandari et al., 2021). However, research is required to identify a new mechanism of the relationship between leader humility and follower creativity (Wang et al., 2017; Mao et al., 2019). Based on the abovementioned discussion, we expect that leader humility will positively influence follower creativity in COVID-19.

*H*_1_: Leader humility is positively related to follower creativity.

### Leader Humility and Follower Self-Efficacy

Self-efficacy plays a motivational role in self-regulation (Bandura, 2001). Efficacy indicated the individual’s confidence in utilizing the cognitive resources, motivation, and courses of action to perform a specific task (Stajkovic and Luthans, 1998). People who are convinced in their efficacy figure out the opportunities rather than focus on risky new ventures (Krueger and Dickson, 1994). Self-efficacy is about one’s belief in shaping and executing a necessary course of action (Bandura, 1997). People with high self-efficacy erect opportunities and try to find out how to surmount institutional impediments (Bandura, 2012). The effect of goal assignment on performance is strengthened when individuals have high self-efficacy (Sue-Chan and Ong, 2002). More perniciously, ability and skill increase the self-efficacy that influences performance goals (Brown et al., 2011).

Individuals with high self-efficacy are more likely to establish higher goals and anticipate actions that lead to performance attainment through intrinsic motivation (Bandura and Locke, 2003; Sitzmann and Yeo, 2013). The social cognitive theory predicts behavior and explains the mechanisms of learning and change (Bandura, 2012). High self-efficacy permits individuals to learn strategies to overcome new challenges and attain challenging goals (Seibert et al., 2017). More preciously, leadership climate predicts individual self-efficacy and group efficacy (Chen and Bliese, 2002). For instance, researchers claimed that leadership styles affect the member self-efficacy on an individual level (Choi et al., 2003).

Efficacy beliefs affect the work-related activities and influence the other functional activities, i.e., decision-making. Implementing a strategy or decision is associated with the decision-making process and self-efficacy (Bandura, 1997). When there is team decision-making, team members show a high level of efficacy as they feel competent (Phillips, 2001). They feel their opinions and skills are valuable for others. So, they exert high-level efficacy, which can influence their decision-making. A leader who allows followers to take part in decision-making may enhance follower self-efficacy. Humble leaders appreciate others and demonstrate open learning and ideas from others (Owens et al., 2013). Hence, leader humility is manifested by exhibiting others’ development and willingness for advice and help. Thus, we expect that leader humility arguments follower self-efficacy.

*H*_2_: Leader humility is positively related to follower self-efficacy.

### Follower Self-Efficacy and Follower Creativity

Personal self-efficacy is related to a person’s positive attitude and capability to perform a different task successfully. The influence of self-efficacy on performance diverges in different circumstances (Stirin Tzur et al., 2016). The literature suggested that self-efficacy can explain why and how there is a relation between cognitive ability and performance (Chen et al., 2001). Furthermore, an external coach affects the participant effectiveness than one’s self or a peer (Sue-Chan and Latham, 2004). Generally, self-efficacy positively influences task performance through the motivational state (Chen et al., 2004) as one’s belief about efficacy regulates cognitive, motivational, and affective function, which enables people to build a productive environment (Bandura, 2001). Researchers recommend a conditional view about self-efficacy; when rewards are high (low), the self-efficacy positively (negatively) influences performance (Stirin Tzur et al., 2016). Ambiguity is a component of negative self-efficacy (Schmidt and DeShon, 2010). The relationship between self-efficacy and performance is contingent on the level of ambiguity (Schmidt and DeShon, 2010). Ambiguity can be reduced by incorporating information at the right time. Timely and exact responses from leaders foster follower self-efficacy.

The social cognitive theory suggests that people with high efficacy anticipate success, which assists as guides for performance (Bandura, 2001). Self-efficacy is a significant predictor of employee performance (Judge and Bono, 2001). Psychological capital contributes toward organizational commitment (Sahoo and Sia, 2015). Self-efficacy is a psychological capital that assists in performing different tasks (Peterson et al., 2011). Individuals having confidence in their abilities accept challenging tasks without concerning uncertainty. Scholars contend that in COVID-19, self-efficacy is positively related to employee performance (Mujeeb et al., 2021). When employees have a high level of self-efficacy, they are more likely to be creative. Self-efficacy contributes to workplace creativity (Appu and Sia, 2017). Further, creative self-efficacy is positively related to employee creativity (Gong et al., 2009). Moreover, creative self-efficacy stimulates the creative process, which helps to develop new and valuable ideas (Bandura, 1997). Accordingly, we propose that follower self-efficacy is positively related to follower creativity.

*H*_3_: Follower self-efficacy is positively related to follower creativity.

### Mediating Role of Follower Self-Efficacy

Generally, researchers have focused on individual creativity and creative efficacy. A workplace event provides viable and indispensable opportunities for creativity. Employee improvisation mediates the relation between the workplace event novelty and creativity (Chen et al., 2020). Drawing on the social cognitive theory, knowledge structure provide a channel that guides how a sub-skill should be integrated, selected, and used for a specific purpose (Bandura, 2001). According to Richter et al. (2012), creative self-efficacy positively impacts creativity when team members have greater shared knowledge of who knows what.

Previous studies have claimed that the influence of leadership styles on followers will be strengthened through an intervening mechanism. Creative self-efficacy mediates the relationship between leadership styles and employee creativity (He et al., 2020). Self-efficacy is a global trait, and it contributes to the operation (Bandura, 2012). However, COVID-19 drastically influences every aspect of life and organization all over the world. We argue that how self-efficacy can be an intervening mechanism during a pandemic. Although previous literature studies provided suggestive evidence that several intervening variables influence leader behavior with creativity. A humble leader gives psychological freedom to the follower to enhances self-efficacy to allow him to be more creative. More recently, self-efficacy mediates the relationship between servant leadership and employee performance under pandemics (Mujeeb et al., 2021). So, based on the abovementioned discussion, we proposed that follower self-efficacy mediates a positive relationship between leader humility and follower creativity.

*H*_4_: Follower self-efficacy mediates the relationship between leader humility and follower creativity.

### Moderating Role of Leader Proactive Personality

A proactive personality secures the psychological resources under stressful COVID-19 conditions (Yi-Feng Chen et al., 2021). Proactive personality is a process by which individual influences the environment (Bateman and Crant, 1993). Proactive personality has been associated with career success (Seibert et al., 1999), and this is a better way by which proactive persons achieve a high level of success (Seibert et al., 2001). People learn what action would be suitable in a specific situation by observing positive and negative outcomes (Bandura, 2001). Similarly, proactive people analyze the situation and take steps to more advantage, which lead to favorable outcomes (i.e., performance). Personality characteristics (i.e., proactive personality) can predict directly and indirectly work engagement, job performance, and mental health (Altura et al., 2020). Though leaders who are proactive supports others proactive behavior as leaders feel the responsibility for construction change and motivate employees to be proactive (Fuller et al., 2015). For instance, proactive personality motivates leader member exchange and voice behavior (Wijaya, 2019).

The social cognitive theory explains that the human mind is proactive and creative (Bandura, 2001). It is more appropriate to say that people think about what action should be taken with changing the situation regardless of whatever alterations may be required. Motivation strengthens the relationship between proactive personality and individual creativity (Farooq et al., 2020). While team members’ proactive personality provides a climate for the innovation that leads to individual creative performance (Xu et al., 2019). The literature provides suggestive evidence that leader behavior could be a boundary condition for leader and follower outcomes. Transformational leadership (i.e., motivation and intellectual stimulation) moderates the relation between educational specialization heterogeneity and team creativity (Shin and Zhou, 2007). Moreover, proactive leader moderates between leaders’ positive behavior and teams for individual creative role identification or individual creative self-efficacy (Wang and Liang, 2020). Yet, the previous literature described a positive relationship between proactive behavior and innovation (Seibert et al., 2001). Even in COVID-19, proactive personality is also associated with performance (Yi-Feng Chen et al., 2021). We proposed that a proactive leader moderates the relation between follower self-efficacy and follower creativity based on extant literature.

*H*_5_: Leader proactive personality will moderate the relationship between follower self-efficacy and follower creativity, such that the relationship of follower self-efficacy on follower creativity will be stronger in the presence of high (vs. low) leader proactive personality.

## Materials and Methods

### Participants and Procedures

To empirically test the hypothesized model, primary data were collected from the followers (i.e., employees) and their immediate supervisor/manager (leader) working in both public and private banks of Pakistan (during COVID-19). The State bank of Pakistan regulate both private and public banks; thus, we targeted these banks. We used convenience sampling for data collection. For collecting the data, we used two questionnaires. The first questionnaire was for followers, and the second one was for leaders. Bank managers were considered as a leader because they overlooked the activities of employees working in the branch. Employees were considered as a follower as they followed the instruction of managers. English language is used to develop both questionnaires, which is easily understandable by the respondents working in the banks of Pakistan. A pilot study was conducted to evaluate the effectiveness of both questionnaires and find out the measures, which are not understandable by respondents. We take consent from all banks’ HR departments to collect the data, especially working in the Punjab province of Pakistan. Then, we emailed questionnaires with a covering letter to those branches, which gave consent to participate in the survey. After 2 weeks, we received a few responses and sent a second email as a reminder and made phone calls to those who did not reply.

Finally, we physically visited the branches to obtain the data. During the COVID-19 period, this study was conducted. We followed standard operating procedures (SOPs), and maintained social distancing and mask wearing. We did not directly interact with each employee. Questionnaires were given to one branch manager; the manager further distributed these questionnaires to the branch’s remaining employees. We targeted 62 branches of different banks and distributed 450 questionnaires among followers and 110 questionnaires among leaders. Finally, 405 useable questionnaires were received from the followers and 87 useable questionnaires from the leaders. It yields a follower’s response rate of 90% and a leader’s response rate of 79%. Concerning followers, the sample data indicate that 73.3% of the respondents are men. Furthermore, the majority of the managers are men in the banking sector (96.5% men). It is noted that employees, as well as managers, are highly educated (Table 1). Around 18% of the data were collected from the first line manager and 82% from non-managerial employees.

**TABLE 1:** Education level of participants.

Education (years)	% of Employees Education	% of Managers Education
12	2	–
14	20	14
15	0.5	1.2
16	67.7	66.3
17	0.5	2.3
18	9.2	15
20	–	1.2
Total	100	100

### Measurement and Scales

To overcome method biases (Podsakoff et al., 2003), the survey data are obtained from two separate sources (followers and their immediate leader). In addition, a neutral scale point is also avoided. The measurement scales and sample items of all variables are provided as follows.

*Follower creativity* is measured by using a 13-item scale adapted from Zhou and George (2001). Employees responded to the 13 items on a 5-point Likert scale ranging from “Never” to “Always.” We adopted a 9-item scale developed by Owens et al. (2013) to measure *leader humility*. Followers responded to the 9 items on a 6-point Likert scale ranging from “strongly disagree” to “strongly agree.” *Self-efficacy* is measured with an 8-item scale developed by Chen et al. (2001). Followers responded to the 8 items on a 5-point scale ranging from “strongly disagree” to “strongly agree.” We measured *leader proactive personality* with a 10-item scale developed by Seibert et al. (1999). Leaders responded on a 6-point Likert scale ranging from “strongly disagree” to “strongly agree.” Keeping in view of extant research, we consider demographic factors as control variables, such as job designation, gender, age, and educational level, tenures in branch and bank.

## Results

To empirically test the hypothesized model, the study utilizes the SPSS and AMOS software. Initially, data cleaning and screening, correlation matrix, and descriptive statistics analyses are conducted. The explanatory factor analysis (EFA) and confirmatory factor analysis (CFA) are performed to confirm the model fitness. Furthermore, Process Macros are used to test the direct, mediating, and moderating relationships. We test the normality of data by using the Kolmogorov–Smirnov test (One-Sample K–S test). Tabachnick and Fidell (2007) suggested that the value of the Kaiser–Meyer–Olkin (KMO) index should be 0.6 or more (ranging from 0 to 1). In the current study, the KMO value is 0.749, and Bartlett’s test is significant as its value of *p* = 0.000. After that, Mahalanobis *D*^2^ test is conducted to find out outliers. Further, reliability is tested through Cronbach’s alpha and composite reliability.

The EFA is conducted to explore the factors that measure the constructs. Further, the items that have poor loading (i.e., loading less than 0.30) are removed. Small coefficient factors are considered to be insignificant (Fabrigar et al., 1999). CFA is conducted to find the best model among different alternate models using AMOS 20. We also run alternate models to find the best model, i.e., 3 factors, 2 factors, and 1 factor. The fit indices reveal that the hypothesized model is the best as compared to alternative models. Model fit indices of the hypothesized model are within acceptable range as well as compared to alternate models [chi-square fit statistics (CMIN)/df = 1.935, goodness-of-fit index (GFI) = 0.853; comparative fix index (CFI) = 0.894; Tucker–Lewis index (TLI) = 0.888, root mean square error of approximation (RMSEA) = 0.048].

The properties of the study variables are normally distributed as the values of SD are less than 1. The average values of all variables are more than 4 (Table 2). Cronbach’s alpha values indicate that scales are reliable and the items in a construct are strongly correlated. The reliabilities of all variables are incredible as the scale coefficients are more than 0.80. The values of Composite reliability of all variables are in the acceptable range (more than 0.70). The alpha and CR values are given in Table 2.

**TABLE 2:** Descriptive statistics and reliability analysis.

Variables	Mean	SD	α	CR
Leader humility	4.61	0.93	0.90	0.90
Follower self-efficacy	4.00	0.64	0.82	0.73
Follower creativity	4.06	0.61	0.89	0.75
Leader proactive personality	4.92	0.75	0.88	0.88

*SD, Standard deviation; α, Cronbach’s alpha; CR, Composite reliability.*

The correlation results (Table 3) reveal that leader humility is significantly related to follower creativity (*r* = 0.433, *p* < 0.01) and follower self-efficacy (*r* = 0.335, *p* < 0.01). Follower self-efficacy is significantly related to follower creativity (*r* = 0.401, *p* < 0.01). The results also indicate that follower self-efficacy has no significant relationship with a leader’s proactive personality. Moreover, follower creativity and leader proactive personality have an insignificance relation (*r* = 0.037).

**TABLE 3:** Correlations matrix.

Variables	1	2	3	4	5	6	7	8
1. Employee gender	1							
2. Employee age	0.221**	1						
3. Formal education	–0.014	−0.306**	1					
4. Tenure branch	0.046	0.242**	–0.085	1				
5. Tenure bank	0.131**	0.658**	−0.289**	0.368**	1			
6. Leader humility	0.007	0.091	0.055	–0.010	0.019	1		
7. Follower self- efficacy	0.021	0.013	0.069	0.029	–0.051	0.335**	1	
8. Follower creativity	0.078	0.031	0.047	0.005	0.022	0.433**	0.401**	1
9. Leader proactive personality	−0.167**	–0.058	–0.036	–0.093	–0.026	0.049	0.039	0.037

**p < 0.01.

Moreover, we used process Macros models (Hayes, 2013) to test our hypothesized model. We used models 1 and 4 to test the mediation and moderation effect (see Table 4). The result reveals that leader humility has a positive significant effect on follower creativity [β = 0.221, *p* < 0.001 (LLCI = 0.162, ULCI = 0.280)]. Further, there is a significant direct effect of leader humility on follower self-efficacy [β = 0.230, *p* < 0.001 (LLCI = 0.166, ULCI = 0.294)]. Thus, Hypotheses 1 and 2 are supported. The findings also exhibit that follower self-efficacy has a positive effect on follower creativity [β = 0.276, *p* < 0.001 (LLCI = 0.190, ULCI = 0.362)]. Therefore, Hypothesis 3 is supported.

**TABLE 4:** Process macros results.

Hypotheses	Path	Direct effect			Indirect effect		
		Beta	LLCI	ULCI	Beta	LLCI	ULCI
H_1_	LH⟶FCR	0.221***	0.162	0.280			
H_2_	LH⟶FSE	0.230***	0.166	0.294			
H_3_	FSE⟶FCR	0.276***	0.190	0.362			
H_4_	LH⟶FCR via FSE				0.063	0.033	0.099
H_5_	FSE⟶FCR Interaction effect of LPP	0.141**	0.013	0.269			

*LH, Leader Humility; FCR, Follower Creativity; FSE, Follower Self-efficacy; LPP, Leader Proactive Personality.*

**p < 0.01; ***p < 0.001.

**FIGURE 2:**
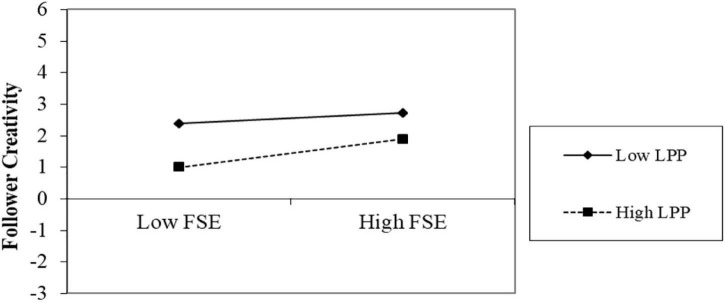
Moderation effect of leader proactive personality.

By using model 4, we tested the mediation effect of follower self-efficacy. The findings support the proposed mediation relation. Follower self-efficacy mediates the relationship between leader humility and follower creativity [β = 0.063, (LLCI = 0.033, ULCI = 0.099)]. Hypothesis 5 depicts that a leader’s proactive personality moderates the relationship between follower self-efficacy and follower creativity. The positive effect of follower self-efficacy on follower creativity is strengthened when leader proactive personality is high [β = 0.141, (LLCI = 0.013, ULCI = 0.269)]. We plotted moderation effects of a leader’s proactive personality in Figure 2.

## Discussion

In the current study, we investigated how humble leadership influenced follower behavior, i.e., follower self-efficacy and follower creativity in the workplace when COVID-19 is at the peak position. We theoretically contend and empirically test that leader humility magnifies follower self-efficacy, which positively affects follower outcomes. This study also attempts to determine a mediating role of follower self-efficacy between leader humility and follower creativity. Furthermore, it is also found that a leader’s proactive personality moderates the effect of follower self-efficacy on follower creativity. The findings of present research support our hypothesized model, demonstrating that humble leaders, directly and indirectly, influence follower behavior. This study analyses the psychological behavior styles such as follower self-efficacy and follower creativity. The findings reveal that leaders are the main component of the social system that influences follower behavior.

Based on the social information theory, we investigated that how leader humility promotes follower creativity. Prior research has identified a positive relation between leader humility and follower creativity, specifically in the healthcare system, i.e., hospital (Wang et al., 2017). We can expect that leaders in other sectors will be humble as doctors. We explore the impact of leader humility on follower creativity in the banking sector. Because the findings represent a positive effect of leader humility on follower creativity, in alignment with previous research (Wang et al., 2017), scholars also reveal that engaging in creativity can buffer depressed situations in COVID-19 (Kapoor and Kaufman, 2020). It should be taken into account for the organization as well as managers for survival.

We explore the relationship between leader humility and follower self-efficacy. Scholars have discussed self-efficacy and its predictors. A humble leader enhances a role of the follower and promotes other expertise. This study empirically proves that leader humility have a positively influence on follower self-efficacy. A humble leader encourages followers to take risks and respond to challenging situations. The findings confirm that bank leaders are humble. Moreover, followers in banks have high self-efficacy. Hence, leader humility is a proven predictor of follower self-efficacy. Findings provide support to the theory (Bandura, 2001). In addition, we predicted that follower self-efficacy could bolster follower creativity. The followers who take the risk and have the confidence to perform a specific task are more likely to be creative. Our findings reveal that follower self-efficacy is positively related to follower creativity in alignment with previous studies (Gong et al., 2009). Hence, it is proven that follower self-efficacy is also a predictor of follower creativity. Moreover, this study tests that follower self-efficacy mediates the relationship between leader humility and follower creativity. Humble leaders enlarge the confidence of their followers to perform challenging tasks. Followers with high efficacy display cognitive resourcefulness, which lead to creativity. It underscores an essential intervening mechanism that describes the association of leader humility with follower creativity. The findings consist of past research showing that efficacy is a valuable intervening mechanism for the relation of a leader with followers (He et al., 2020). It is noted that self-efficacy is an intervening mechanism under COVID-19 (AlZgool et al., 2020).

Finally, we explore a leader’s proactive personality as a critical moderator and empirically find that a leader’s proactive personality positively moderates the relationship between follower self-efficacy and follower creativity. Nevertheless, researchers pay attention to a leader’s proactive personality as a moderator for leader humility and leadership styles (Chiu et al., 2016). We found that leader proactive personality is a boundary condition between the relation of follower self-efficacy and follower creativity. Nowadays, a proactive personality has become more critical for every organization. Proactive people can best analyze the environment in the best way. A proactive person can control COVID-19 crises by focusing on motivation resources (Yi-Feng Chen et al., 2021).

The study contributes to the literature in several ways. This study responds to a recent call for further investigating the relation between leader humility and follower creativity (Mao et al., 2019). It is an extension of the literature on leader humility by explaining the role of self-efficacy. The present study provides a motivational mechanism and empirically supports that a humble leader enhances follower creativity through self-efficacy even in the COVID-19 pandemic. Furthermore, several studies were conducted on the concept of leader humility and leader proactive behavior. This study finds out how a leader’s proactive personality positively moderates the relationship between follower self-efficacy and follower creativity. This study provides insights that humble leaders and proactive leaders are beneficial for the organization and try to identify opportunities and influence the environment through an innovation. The study highlights the importance of leader humility for creativity during COVID-19. Humble leaders express open learning and new and unique ideas from others (Owens et al., 2013). In that way, a humble leader cultivates follower creativity, which may articulate the innovation culture in the services industry (i.e., banking sector).

### Implications of This Study

Theoretically, the current study describes new insights toward the literature on leader humility and its boundless favorable influences on follower behavior during COVID-19. This study is an extension of the literature on leadership that leaders may enhance follower creativity based on social information processing. Furthermore, it advances the literature on leader humility by focusing on individuals, improving organizational outcomes, and leading toward innovation and social sustainability. Based on the social cognitive theory, humble leaders build interpersonal relations and appreciate others, enhancing follower outcomes. For instance, leader humility augments follower self-efficacy. Moreover, it pays attention to follower behavior instead of focusing on organizational outcomes. This research can be fruitful for scholars to further explore the unrevealed aspect of leader humility. This study also contributes to investigation of the intervening mechanism of follower self-efficacy. However, creative self-efficacy is proven to be a motivational mechanism for leader and follower relationships (He et al., 2020).

We empirically proved that follower self-efficacy strengthens the relation between leader humility and follower creativity. This study again proves that self-efficacy is an intervening mechanism during COVID-19. In a stressful situation, self-efficacy works as a motivational factor. It also shows the importance of leader humility how humble leaders maintain their followers’ capability and confidence to perform a task in a pandemic. COVID-19 creates a depressing environment everywhere and causes mental health issues. Leader humility can deal with such a depressing situation. When leaders are proactive, the relation between follower self-efficacy and follower creativity is stronger. It extends the literature by incorporating that a proactive person not only controls the dynamic environment but also help others to cope with uncertainty, such as proactive person shapes the climate, which encourages follower self-efficacy, which in turn influence follower creativity. Hence, it is proven that a leader’s proactive personality influences his personality and improves his followers.

According to the social cognitive theory, proactive people can control the environment. In COVID-19, it is a big challenge for every organization how to deal with this uncertain environment. This study reveals that a proactive person can control business activities in a pandemic situation. A proactive person can secure the psychological resources and their strength. Practically, this study has various implications for practitioners and policymakers of the service sector, particularly the banking sector. The humble leader can achieve pecuniary advantages and human, social, and ethical outcomes as humility is a moral issue. In the vivacious environment, the main objective is to gain a financial advantage and consider ethical matters as humble leaders promote them. The organization should embrace an environment, which encourages leader humility and leader proactive behavior. Leaders or managers should support an environment that allows the follower to give unique and novel ideas. This study embarks that managers should promote leader–follower self-efficacy.

No doubt, leader humility has a positive impact on creativity. However, there is a need to take advantage of leader humility using different tools. Organizations should arrange a training program for a leader for promoting leader humility and proactive personality. The human mind is proactive and creative; when organizations give proper training, they will take advantage of proactive and humble leaders at the right time. Global competition stimulates organizations to be innovative and socially sustainable. In a vibrant environment, policymakers should pay attention to competition for survival. Furthermore, the COVID-19 pandemic drastically affects the organizations, and employees are critical for survival in the work environment. When followers have the confidence to perform a specific task and have the authority to take a risk, this leads to follower creativity. In the workplace, the organization should promote creativity for dealing with an uncertain business environment. A humble leader endorses follower self-efficacy, which leads to follower creativity. Hence, the findings emphasize that the management should develop a recruitment policy that promotes employee creativity, innovation, and social sustainability regardless of the restrictions (i.e., lockdowns and social distancing).

## Conclusion

By adopting the social cognitive and social information processing theory, this study provides evidence that leader humility, directly and indirectly, influences follower behavior during the coronavirus pandemic, such as leader humility has a positive impact on follower self-efficacy and creativity. This research also demonstrated a relation of follower self-efficacy with follower creativity. We explore the intervening mechanism of follower self-efficacy. Further, we describe that a leader’s proactive personality moderates the relationship between follower self-efficacy and follower creativity. We empirically provide evidence that leader humility and a leader’s proactive personality positively impact followers and organizations in panic environments (the COVID-19 pandemic). These findings highlight the importance of leader humility and a leader’s proactive behavior for creativity at the workplace. Organizations need to focus not only on the leader but also on the follower to gain a competitive advantage in a dynamic situation.

### Limitations and Research Directions

Regardless of several implications, strengths, and contributions to the literature, there are also some limitations. *First*, this study focused on the service sector (i.e., banking sector) only. Hence, the results cannot be generalized for the manufacturing industry. More specifically, the banking sector cannot measure creativity as measured in the high-tech industry. However, future research may be conducted in the industries that are highly dynamic and innovative (i.e., technology industry). *Second*, in this study, many aspects remained unexplained (i.e., hierarchical levels). For instance, the CEO may impose a strategy that limits the leader power. Researchers are unable to rule out the probability of the influence of organization hierarchical levels. Future research can consider the effect of the hierarchical level on follower behavior, i.e., visionary leadership. *Third*, we collected the data at once (i.e., cross-sectional design). However, a cross-sectional design may not support casual relations between leader humility and follower creativity *via* follower self-efficacy. In the future, researchers can explore the causal effect of leader humility on followers’ behavior and a moderating effect of proactive behavior using a longitudinal design. *Finally*, in this study, follower self-efficacy is used as a mediator. We focused on the humility leadership influences and creativity *via* follower self-efficacy. The result indicates that a mediation path is partially supported. Future research can use creative self-efficacy as a mediator that may attenuate or accentuate the effect of leader humility on follower creativity.

## Data Availability Statement

The raw data supporting the conclusions of this article will be made available by the authors, without undue reservation.

## Ethics Statement

Ethical review and approval was not required for the study on human participants in accordance with the local legislation and institutional requirements. The patients/participants provided their written informed consent to participate in this study.

## Author Contributions

All authors listed have made a substantial, direct, and intellectual contribution to the work, and approved it for publication.

## Conflict of Interest

The authors declare that the research was conducted in the absence of any commercial or financial relationships that could be construed as a potential conflict of interest.

## Publisher’s Note

All claims expressed in this article are solely those of the authors and do not necessarily represent those of their affiliated organizations, or those of the publisher, the editors and the reviewers. Any product that may be evaluated in this article, or claim that may be made by its manufacturer, is not guaranteed or endorsed by the publisher.
